# Transperineal ultrasound-guided prostate biopsy: what the radiologist needs to know

**DOI:** 10.1186/s13244-022-01210-x

**Published:** 2022-04-25

**Authors:** Jack Power, Mark Murphy, Barry Hutchinson, Daragh Murphy, Michelle McNicholas, Kiaran O’Malley, John Murray, Carmel Cronin

**Affiliations:** 1 Radiology Department, Mater Misericordiae University Hospital, Eccles St, Dublin 7, Ireland; 2 School of Medicine, University College Dublin (UCD), Dublin, Ireland; 3 Mater Private Hospital, Dublin, Ireland

**Keywords:** Prostate, Biopsy, Radiology, Prostatic neoplasms

## Abstract

Transperineal ultrasound-guided (TP) prostate biopsy has been shown to significantly decrease the risk of post-procedural sepsis when compared to transrectal ultrasound-guided (TRUS) prostate biopsy. With guidance from the European Urology Association favouring adoption of a TP biopsy route, it is clear that, despite being a more technically challenging procedure, TP biopsy in an outpatient setting will replace TRUS biopsy. This paper gives the reader a succinct summary of outpatient transperineal prostate biopsy under local anaesthetic utilising a free-hand ultrasound technique. Patient preparation and consent process is outlined. A comprehensive pictorial review of the procedure, pitfalls and common post-procedural outcomes is presented. This paper provides a framework and guide for those wishing to adopt the transperineal approach under local anaesthetic.

## Key points

- Transperineal ultrasound-guided (TP) biopsy reduces the risk of post-procedural sepsis versus transrectal ultrasound-guided (TRUS) biopsy.
- TP biopsy can be performed safely as an outpatient, under local anaesthetic.
- TP biopsy can successfully replace TRUS biopsy in diagnosis of prostate cancer.

## Background

Transrectal ultrasound-guided prostate (TRUS) biopsy is the most common method of prostate biopsy worldwide [1]. TRUS biopsy has long been associated with a significant risk of sepsis, with 12 biopsies usually taken through an inherently contaminated ‘transfaecal’ route [2]. In 2013, a Canadian study of 75,190 men found a fourfold increased risk of hospitalisation following TRUS biopsy over the decade 1996–2005 [3]. This risk of sepsis post-TRUS biopsy has been increasing over time due to increasing rates of multi-drug resistant bacteria with extended-spectrum β-lactamase (ESBL) and quinolone-resistant bacteria now routinely seen in rectal flora [4]. Reflecting this, a Canadian study reported that the incidence of infection had significantly increased from 0.52% in 2002–2009 to 2.15% in 2010–2011 (*p* < 0.001) [5]. This increasing risk of sepsis has also been seen in studies in Europe [6].

Transperineal ultrasound-guided prostate (TP) biopsy is performed with the ultrasound probe in the rectum and the biopsy samples taken through the perineum. TP biopsy is considered a ‘clean’ procedure, compared to TRUS biopsy being a ‘contaminated’ procedure. TP biopsy has traditionally been performed under general anaesthetic and ‘Template biopsy sampling’ has been employed in this setting with the number of cores varying between 20 and 45 [7]. Transperineal biopsy has been shown to have significantly reduced rates of post-procedural infection [8–12]. A systematic review and meta-analysis performed in 2019 showed no difference in diagnostic accuracy between TRUS and TP biopsy (RR 0.94, 95% CI 0.81–1.10), while showing a significant protection from rectal bleeding and fever in the transperineal biopsy populations (RR = 0.02, 95% CI 0.01–0.06 and RR = 0.26, 95% CI 0.14–0.28, respectively) [10]. This review involved randomised control trials (RCT) comparing the two routes of biopsy, however, the TP biopsy populations involved were primarily those performed under general anaesthetic with a template biopsy technique. This involved multiple punctures to the skin, usually in selected patients who had already undergone TRUS biopsy with either negative results and high clinical suspicion or a history of prior sepsis.

TP biopsy under local anaesthetic has the potential to be offered to all patients in an outpatient day-case setting. Szabo performed a literature review in February 2021 looking at all papers (RCT, case–control, case series) that involved ‘free-hand’ TP biopsy performed under local anaesthetic. In his review of over 7,000 biopsies, the pooled data showed 0 cases of sepsis (0.0% [0/7396]) [13]. With encouraging studies, such as those above, TP biopsy under local anaesthetic is becoming popularised. Papers such as ‘TREXIT 2020’ have highlighted the benefits of moving from a contaminated to a clean procedure and have implored radiology and urology colleagues to join them in this transition [14]. In fact, Pilatz et al. state in the recent European Association of Urology position paper on the prevention of infectious complications following prostate Biopsy that *‘Available evidence highlights that it is time for the urological community to switch from a transrectal to a transperineal PB approach despite any possible logistical challenges’* [15]*.’*

We provide an in-depth guide for Radiologists wishing to perform this important procedure from patient consent to post-procedure care.

### Consent

As with all forms of patient consent, informed consent is key to a successful outcome. Patients due to undergo TP biopsy should receive a patient information leaflet that details the procedure when they attend the urology outpatient clinic at the time of referral for biopsy. Contact details for dedicated prostate cancer specialist nurses who can help answer any questions would also be helpful. On the day of the procedure, a radiologist details the procedure again and describes the risks, benefits and alternatives, after which informed consent is obtained.

Important information in the consent process for TP biopsy include the risk of post-procedural sepsis. Traditionally, this has been quoted as 0.1% or 1 in 1000, however, these figures relate to experience with Template Prostate TP Biopsy and as stated above, the risk of infection and sepsis related to TP Biopsy under local anaesthetic was much lower (0%) as found by Szabo et al. (0.0% [0/7396]) [13]. The risk of post-procedural urinary tract infection quoted to patients is 1 in 100 [13]. The risk of acute urinary retention is 0.1–2.1% [13], with patients encouraged to drink plenty of fluids after the procedure to help prevent clot retention. Patients are advised that blood-tinged urine or semen can be commonly seen after the procedure and is self-limiting in the majority of cases. Blood in the urine can last for up to 10 days and up to 6 weeks in the semen. We have found from patient feedback that perineal pain after the biopsy is common and our patients are advised that they may experience some perineal pain or bruising. They are reassured that this is to be expected and that simple analgesia may be used to ease their symptoms.

### Equipment (Table 1)

**Table 1:** Equipment required to perform a transperineal prostate biopsy

Equipment	Comment
Mefix self-adhesive fabric tape (SCA Mölnlycke Ltd) 5 cm	2 Strips—to elevate scrotum
Razor	To ensure aseptic technique
Chloraprep skin antiseptic (2% chlorhexidine gluconate + 70% isopropyl alcohol [CHG + IPA] in a 3.0-mL applicator) (Medi-Flex Hospital Products, Inc., Overland Park, Kan)	Time given to allow fully dry
Xylocaine 10 mg/ delivered dose mucosal spray	Applied to perineal skin for added anaesthesia
1 × 25 G needle and 1 × 20 G spinal needle	25 G needle for skin and 20 G needle for deeper infiltration
Lidocaine	
Instillagel	Inserted per rectum
Condom type cover for Covering Rectal Ultrasound probe	
1 × 11 cm 17 G Temno Introducer Needle and 1 × 16 cm 18 G Biopsy Needle Gun	For taller or larger patients a longer system may be required (15 cm introducer/20 cm needle)
Specimen Containers	Prelabelled
Opsite Spray to skin after procedure	Dressings are not suitable for perineum

**Fig. 1:**
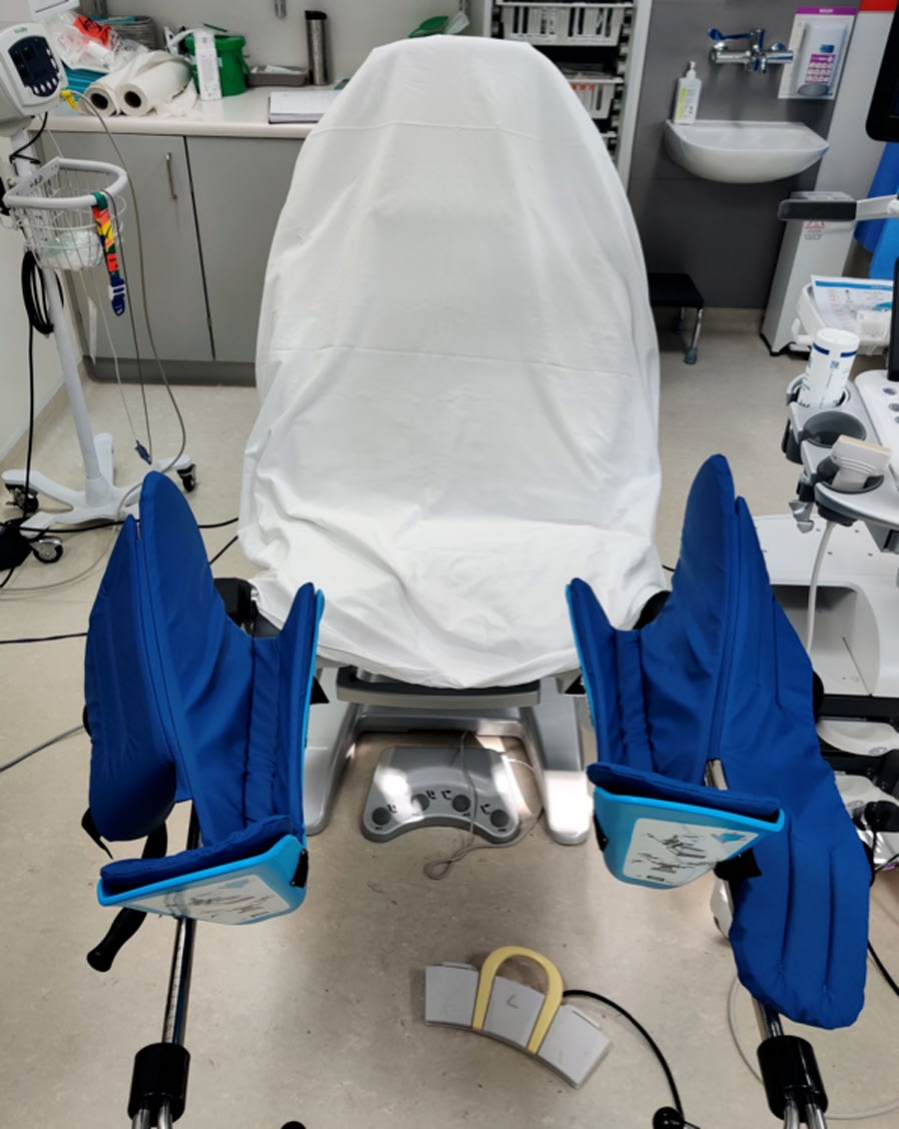
Arco Matic 300 M electrically operated lithotomy Chair with fin type padded boot stirrups (Schmitz, Germany)

**Fig. 2:**
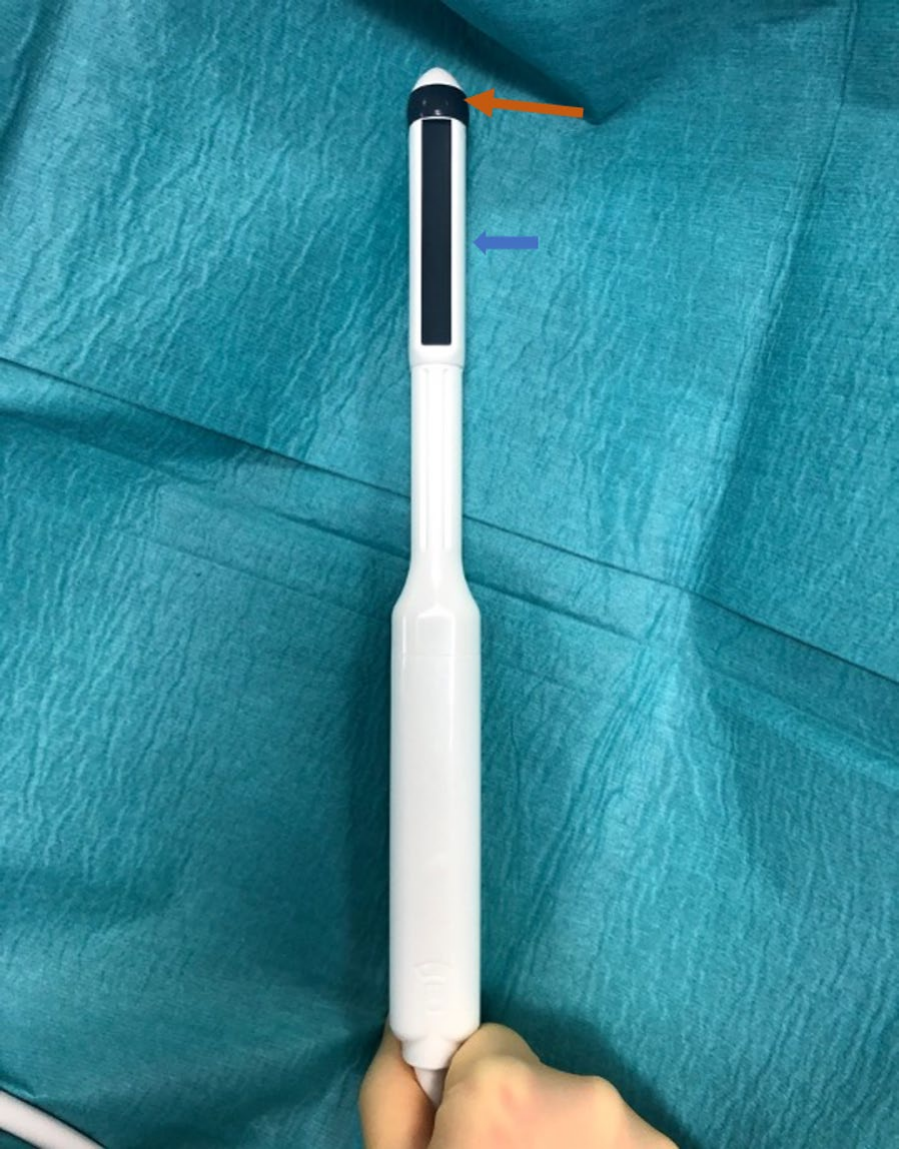
Hitachi Arietta 65 Ultrasound machine with biplane transrectal probe. The orange arrow represents the axial transducer, and the blue arrow, the sagittal transducer

**Fig. 3:**
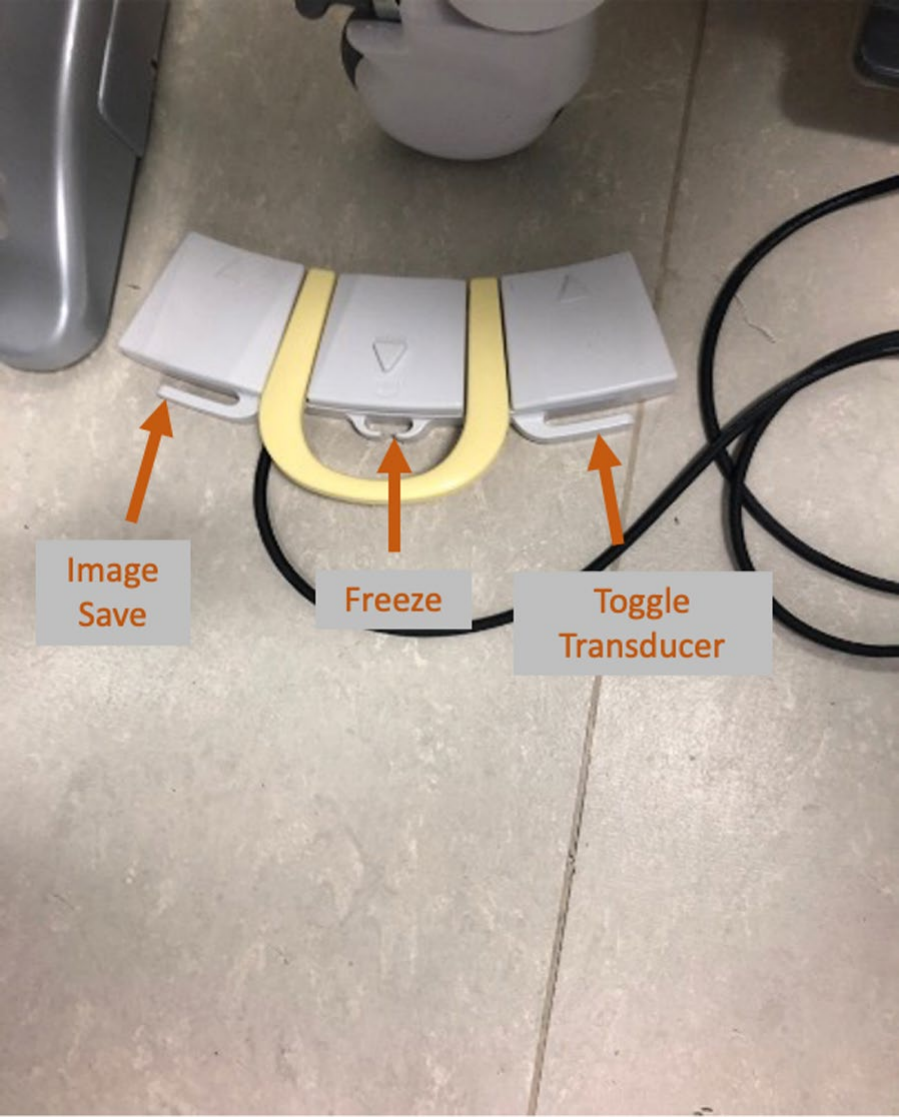
Three gang footswitch (Hitachi Medical Systems, Germany)

We use an Arco Matic 300 M electrically operated lithotomy Chair with fin type padded boot stirrups (Schmitz, Germany) (Fig. 1) and a Hitachi Arietta 65 Ultrasound machine with biplane transrectal probe (Fig. 2) and three gang footswitch (Hitachi Medical Systems, Germany) (Fig. 3).

### Patient preparation and setup

Patients are fasting from food for four hours in case sedation is required. Patient medications are checked prior to booking. Anticoagulation and anti-platelet medications are stopped as per department protocol (Table 2). Aspirin may be continued. Prior to the procedure, medications are again checked and patient imaging is reviewed. In our institution, a bi-parametric prostate MRI is performed prior to almost all prostate biopsies as per NCCP guidelines (National Cancer Control Programme). This is reviewed prior to the procedure, along with any prior TRUS or TP biopsy images and histology results. At the time of biopsy, the entire gland is reviewed with transrectal ultrasound, looking for an ultrasound correlate for any suspicious lesions identified at the time of MRI. This method of targeted biopsy is known as cognitive fusion biopsy and is a proven method of biopsy for TRUS biopsy [16] with studies more recently showing it to be effective via the TP route [17, 18].

**Table 2:** Pre-procedural anti-platelet and anticoagulation management

Medication	Discontinue yes/no	Timing of last dose before procedure if discontinuing	Timing of first dose after day of procedure
Aspirin	No		
Clopidogrel Ticagrelor	Yes	6 days	Day + 1
Prasugrel	Yes	8 days	Day + 1
Warfarin	Yes	6 days (INR check 24 h prior)	Day 0 (evening)
Low molecular weight heparin (LMWH)	Yes	Prophylactic > 12 h Therapeutic > 24 h	Day + 1
(IV) Unfractionated heparin	Yes	Infusion to stop 4 h prior (PTT < 50 s)	6 h
Dabigatran	Yes	GFR > 50- 2 days GFR < 50 – 3 days	Day + 2
Rivaroxaban Apixiban	Yes	2 days	Day + 2
Fondaparinux	Yes	Prophylactic > 24 h Therapeutic > 48 h	Day + 1 Day + 2

### Antibiotics

Currently, there are not enough randomised controlled trials assessing the requirement for prophylactic antibiotics in TP biopsy under LA [12] and the use of antibiotics varies amongst different institutions [13]. In our institution, Oral Amoxicillin/clavulanic acid (500/125 mg) is given as antibiotic prophylaxis an hour prior to the procedure. This is to cover for skin contaminants.

### Patient positioning

Firstly, patients are seated in the modified lithotomy bed. This has moveable stirrups that allows optimum positioning both for patient comfort and biopsy route access. Prior to positioning, it is important to inquire about arthritis and prior surgery at the hips, knees or back to insure a safe and comfortable position can found as this will impact on patients’ tolerance of positioning and the procedure overall. A comfortable position will relax the perineal musculature, some of which inserts into the lesser trochanter of the hip. The legs are elevated, abducted, and supported in stirrups, with the buttocks even and symmetrical, protruding slightly further than the lower end of the table. During elevation, the feet are held in one hand and the lower part of the leg in the other, while the legs are slowly flexed. To prevent hip pain or muscle strain from the exaggerated range of motion, the legs should be raised and lowered slowly and simultaneously once in the stirrups.

Relaxation of the perineal muscles is important to permit movement of the probe within the rectum and to allow the introducer needle pass easily through to the levator muscles. Some external rotation of the hips assists in relaxing the upper thigh muscles.

### Analgesia

Along with patient observation and delivery of nursing care, radiology nursing staff can play an essential role throughout this procedure through conversation and distraction, almost always obviating intravenous sedation. If intravenous sedation is required, we use a titrated combination of Fentanyl and Midazolam delivered in small aliquots.

The strength and volume of local anaesthetic vary widely in the literature [13]. In the review paper by Szabo, the most common anaesthetic found to be used was 1% lidocaine. The volume used varied between 10 and 40 mL across the different studies [17–25]. Other studies reported using 10–20 mL of 2% lidocaine, including the study with the largest sample size (n = 3000) [26–29]. Other forms of analgesia used include a combination of 1% lidocaine with epinephrine to the skin with 1% lidocaine to the deeper tissues [30], 20 mL of 2% mepivacaine [31, 32] or a combination of 0.5% bupivacaine plus 2% mepivacaine [33].

### Procedure

Once the correct position is found, the scrotum is taped back with 2 Mefix strips and a drape is placed over the patients’ knees. We employ the ‘scrotal scoop’ technique, allowing the patient to cover and scoop up the genitalia with their gown and then tape the scrotum in place on the lower abdomen/upper thighs with two lengths of Mefix tape, endeavouring not to apply the tape directly to the scrotal skin. Shaving of the perineum is performed, if required, and the skin is prepped with chlorhexidine.

Instalagel is inserted into the anus and rectum, followed by a biplanar transrectal ultrasound probe which is covered with a latex-free probe cover and copious amounts of sterile coupling gel. A 3-way foot pedal allows for toggling between transverse and longitudinal views, freezing the image and saving an image. The three way foot switch is a very useful asset, removing the need to touch the keyboard or screen to change planes or to capture an image.

**Fig. 4:**
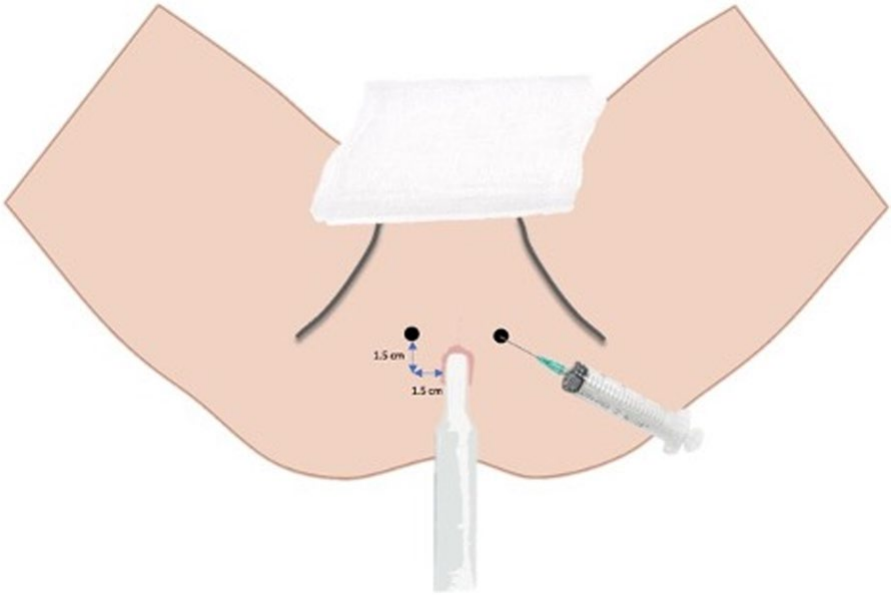
Local anaesthetic and co-axial needle access points

**Fig. 5:**
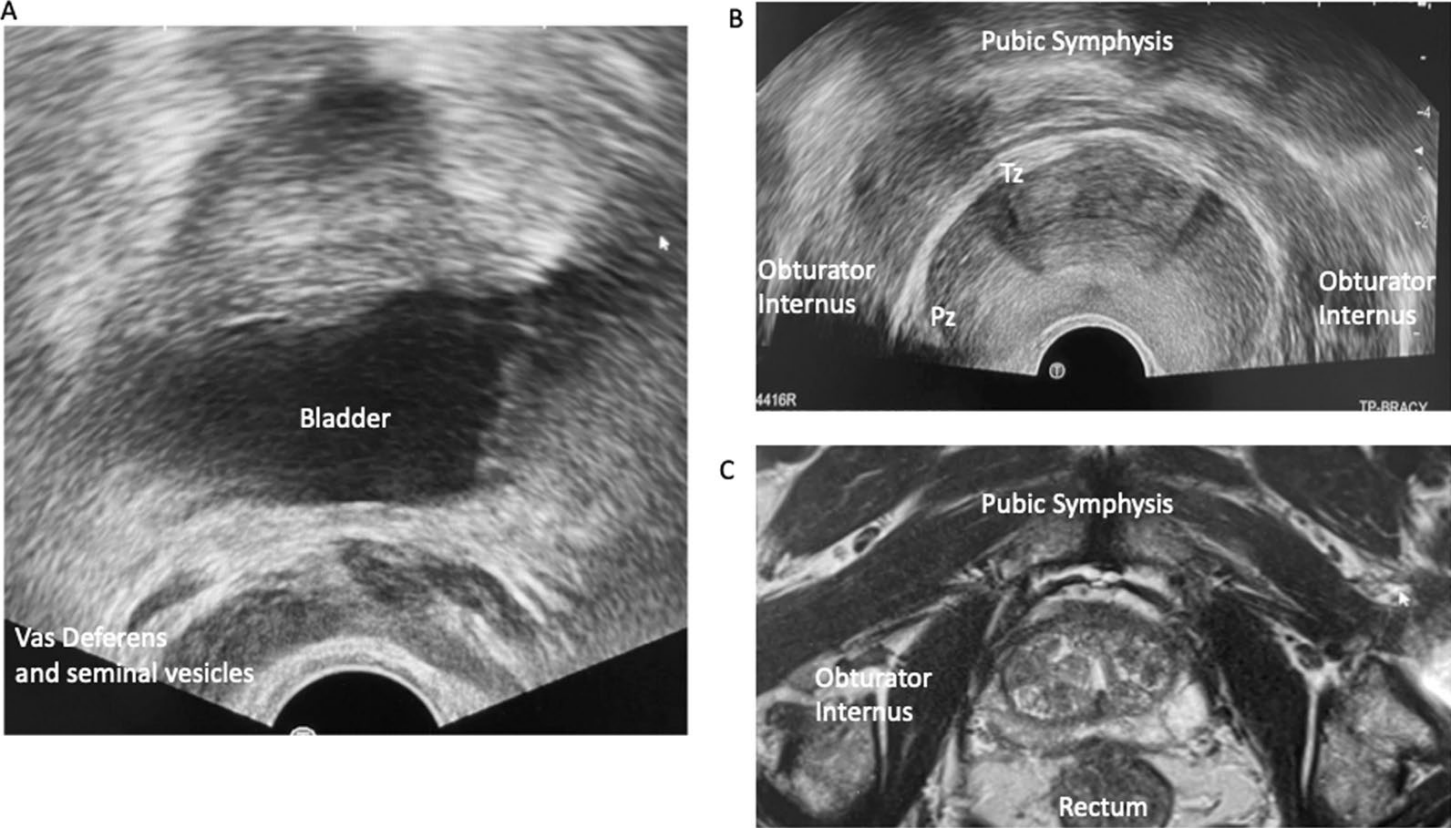
**A** shows axial ultrasound anatomy superior to the prostate. **B** and **C** show the gland and surrounding structures at its mid-level on ultrasound and T2-weighted MRI, respectively. The peripheral zone (Pz) and transitional zone (Tz) can be identified in **B**

**Fig. 6:**
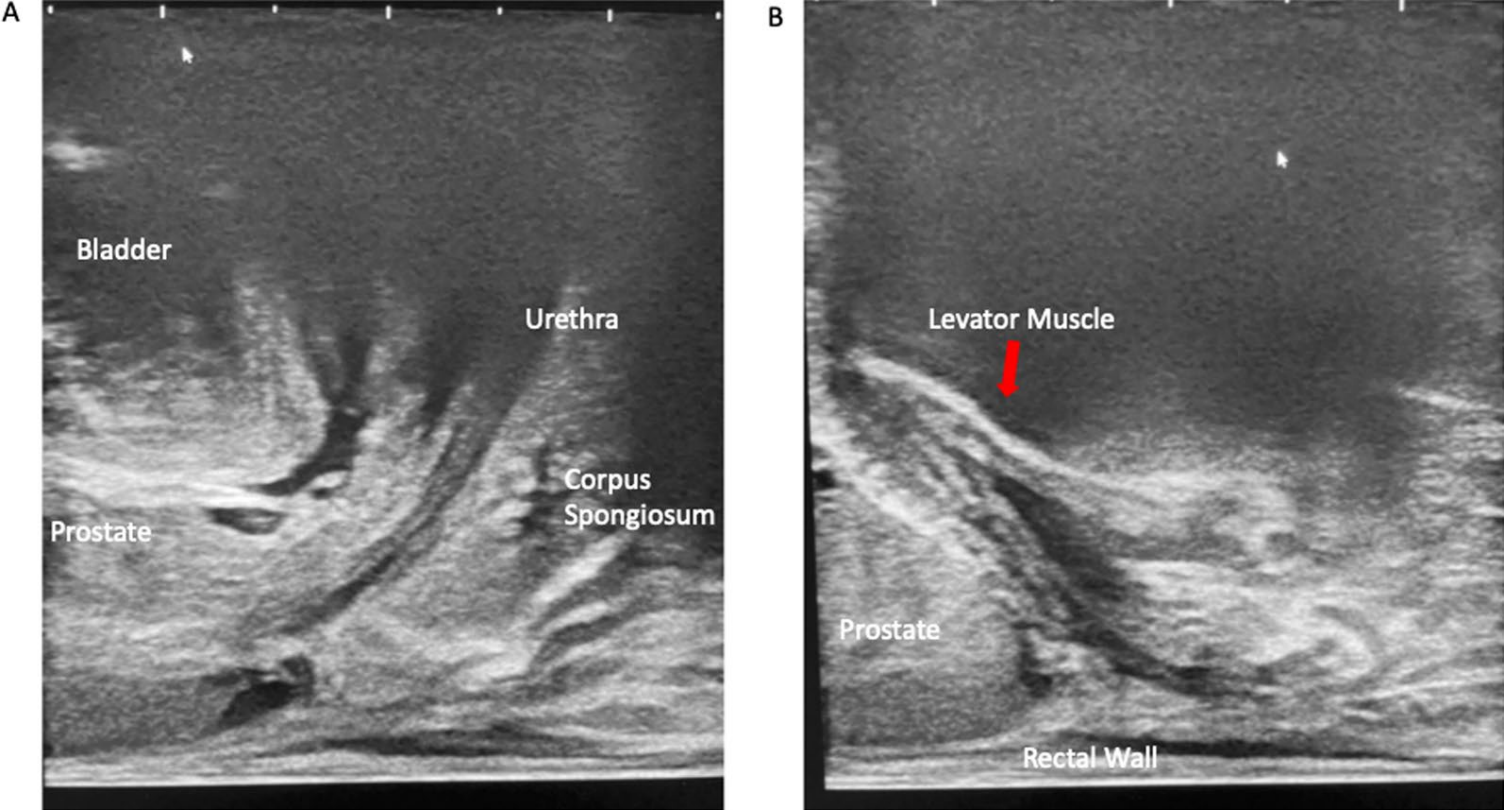
**A** shows sagittal ultrasound anatomy at the mid-portion of the gland, with the presence of the urethra helping confirm a midline location. **B** shows anatomy laterally, with the levator muscles located on either side of the gland

Once the skin has been allowed to dry, local anaesthetic is administered to the perineal skin (via a 25 G needle). This is infiltrated into the skin approximately 1 cm anterior and lateral to the anterior anal margin on either side (Fig. 4). While the anaesthesia is taking effect, the prostate is imaged in both transverse and longitudinal planes to assess the normal pelvic anatomy (Figs. 5 and 6) and to try to identify a correlate for any clinically suspicious lesions identified on pre-procedural MRI. Because the transverse transducer is at the tip of the probe and the longitudinal transducer is closer to the handle of the probe, the probe must be moved in and out when switching from transverse to longitudinal views. The probe must be rotated left and right in the sagittal view to image/visualise the lateral part of the gland. Volume measurements are taken which may be of use for treatment planning (e.g. if brachytherapy is being considered).

**Fig. 7:**
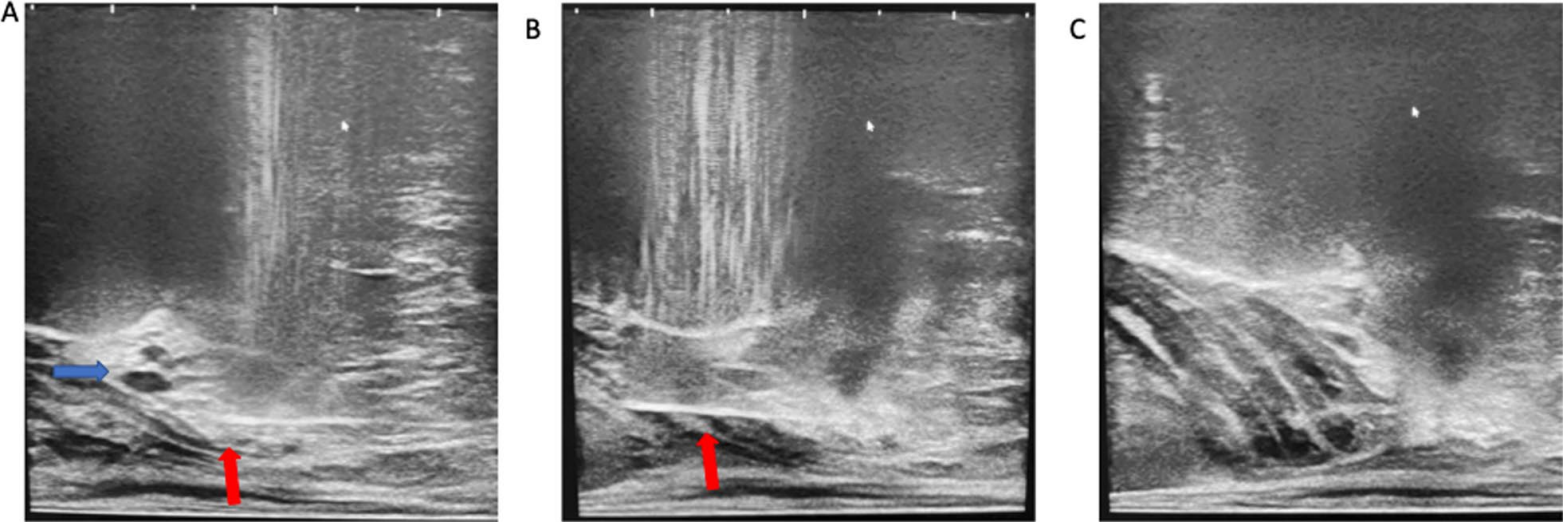
This shows local anaesthetic administration to the levator ani muscle complex and periprostatic space. In **A** and **B**, the red arrow is pointed to the 20-gauge spinal needle used for local anaesthetic administration. The blue arrow in **A** shows small periprostatic vessels, which are avoided. **C** shows an expanded levator ani complex post-local anaesthetic administration

Once adequate superficial local anaesthesia has been achieved, a 9 cm 22 G spinal needle is introduced under longitudinal ultrasound guidance, to administer local anaesthetic to the levator ani muscle complex (Fig. 7) as well as the space between the apex of the prostate gland, bathing the neurovascular bundles of the prostate capsule. Care is taken to avoid the urethra (Fig. 6), the penile bulb, and small perineal vessels (Fig. 7, blue arrow) which are seen in the midline and the introducer needle passes lateral to them in a para-sagittal trajectory to the prostate. Intravascular injection can be avoided by careful aspiration prior to injection.

**Fig. 8:**
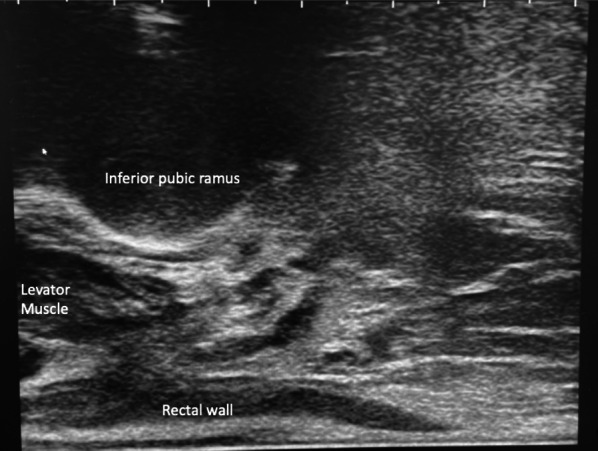
Identification of a low-lying inferior pubic ramus allows for additional local anaesthetic administration to the associated musculotendinous structure

On occasion depending on the variations in pelvic anatomy and pelvic bone tilt, the interior pubic ramus may be prominent leaving only a small window through the ischiorectal fat to the levator muscle and prostate. When this occurs if the needle passes close to the bone and adjacent musculotendinous structure, additional pain may be experienced by the patient. Knowledge and recognition of a low lying inferior pubic ramus allows the radiologist to administer additional anaesthesia to this region (Fig. 8).

**Fig. 9:**
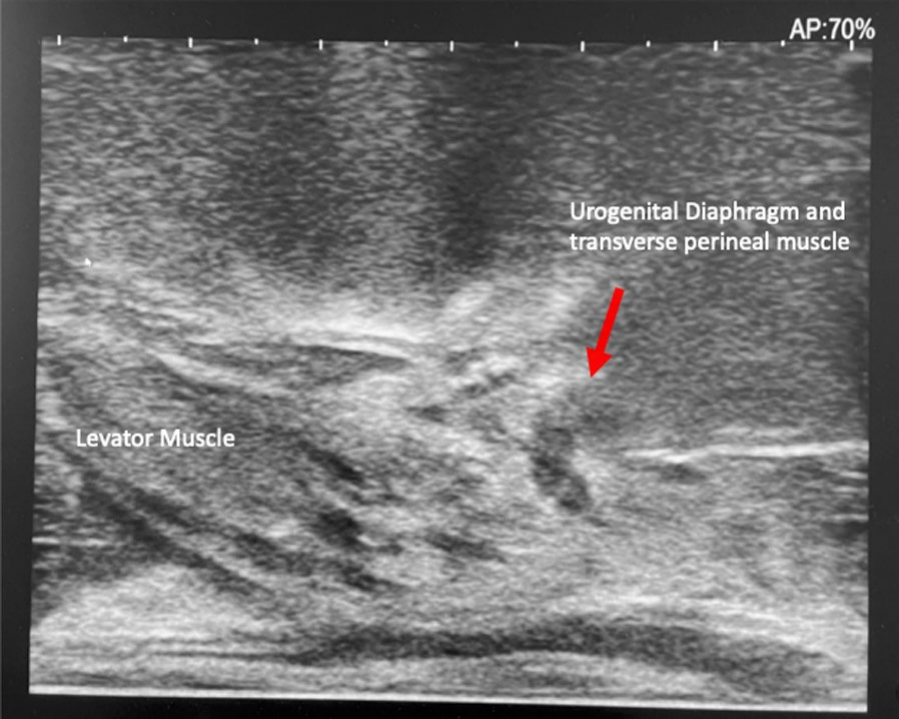
Prominent urogenital diaphragm in a younger patient

**Fig. 10:**
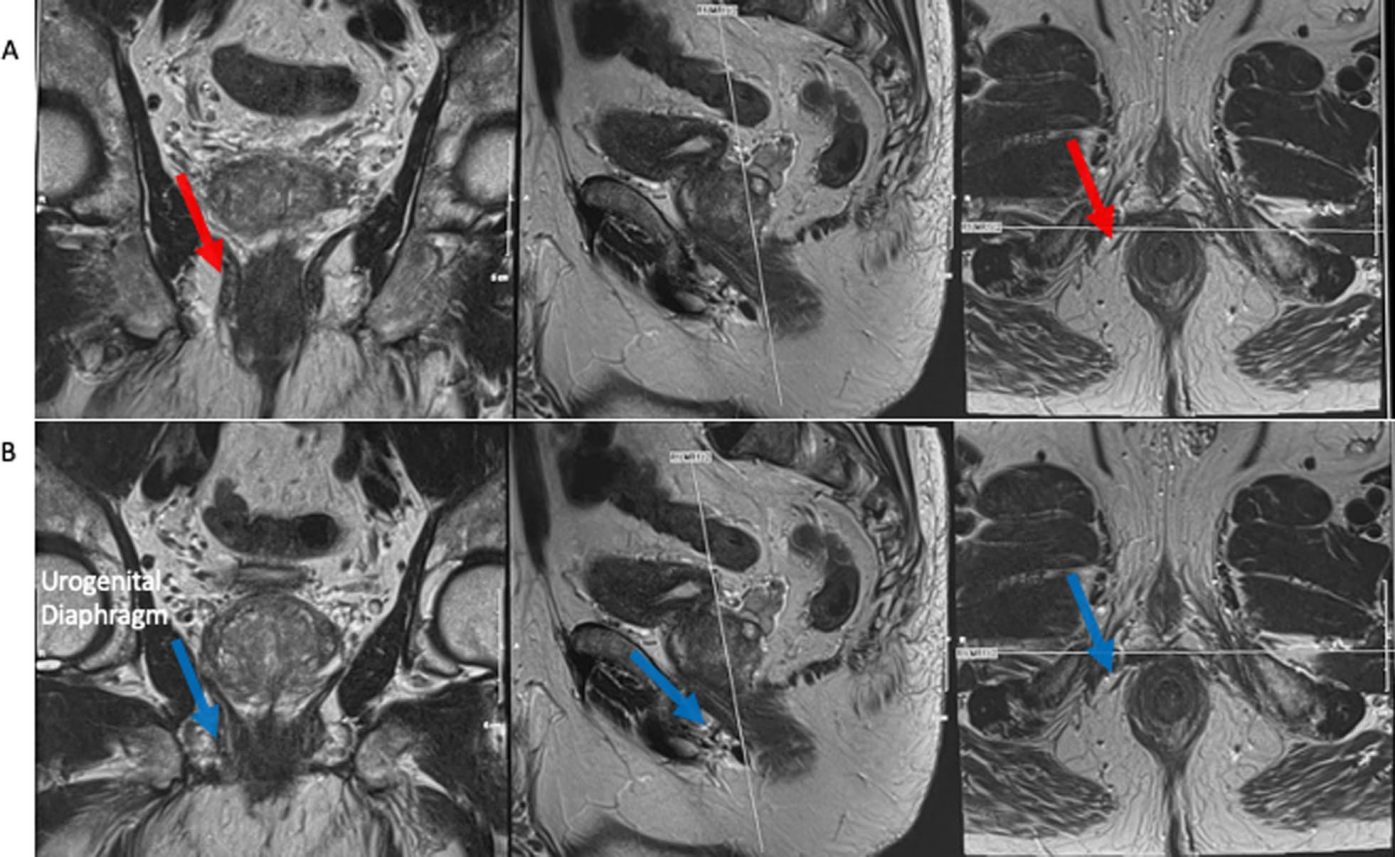
Identification of a prominent urogenital diaphragm on pre-procedure MRI. **A** shows a more posterior location along the urogenital diaphragm (red arrows), with **B** showing a more anterior location (blue arrows)

**Fig. 11:**
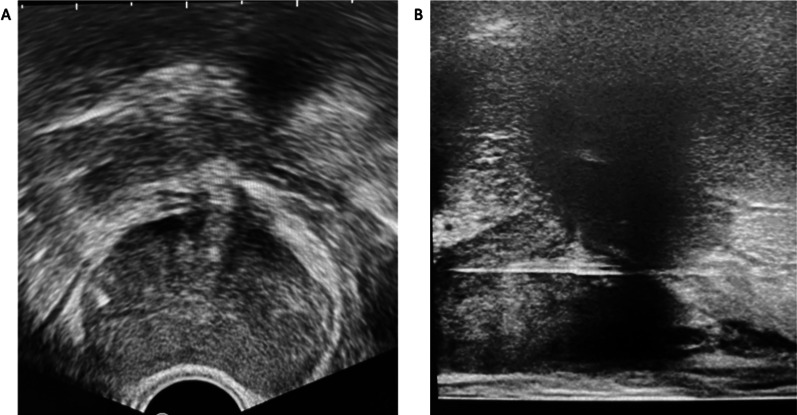
Toggling between axial (**A**) and sagittal (**B**) views can help confirm position of the biopsy needle

**Fig. 12:**
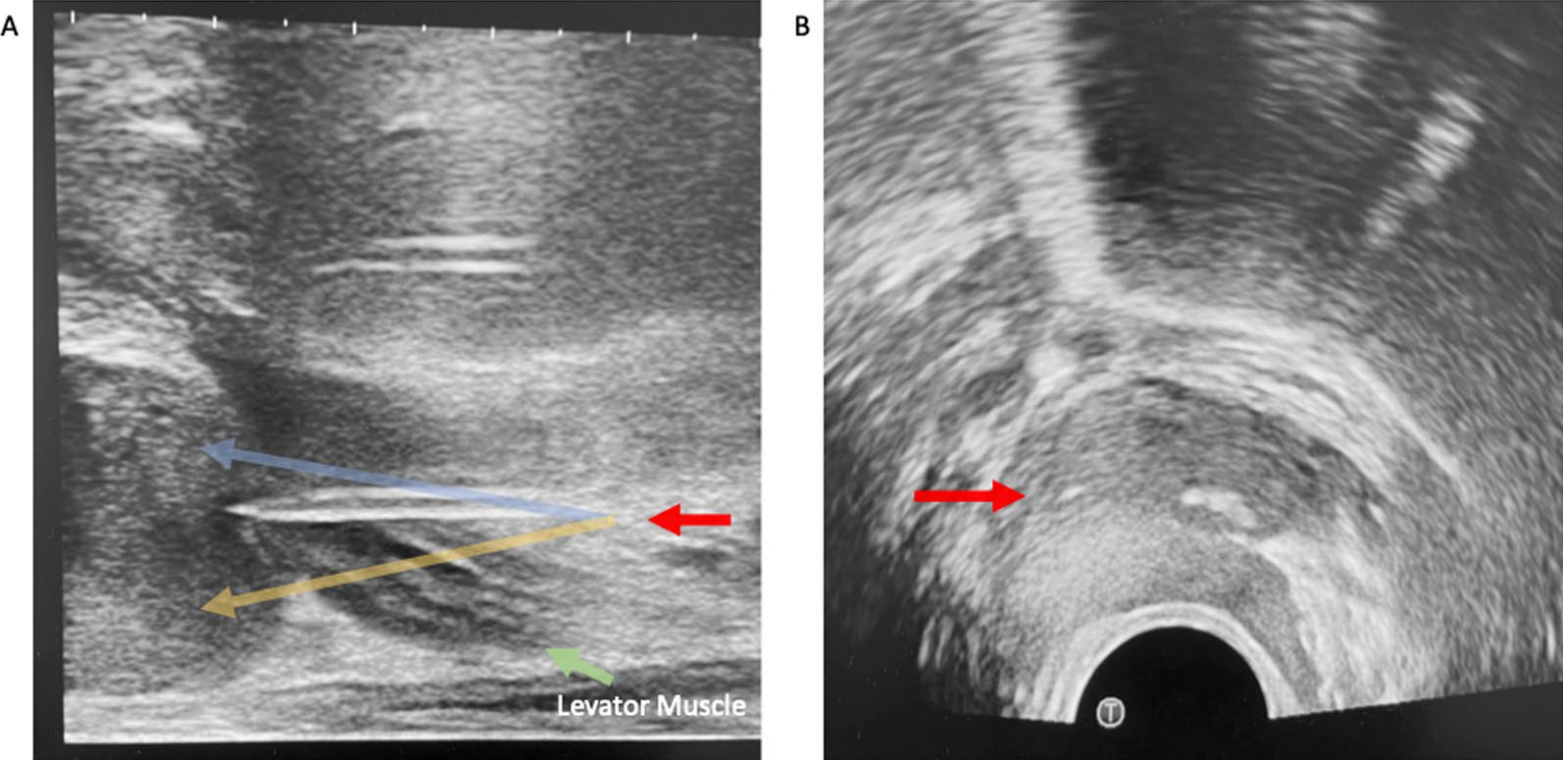
In **A**, the red arrow shows the biopsy needle within the mid-portion of the gland. By angling the head of the probe superiorly (operators hand towards the floor), the anterior gland may be accessed (blue arrow). By angling the probe inferiorly (operators hand towards the ceiling), the posterior gland may be accessed (yellow arrow). **B** shows the tip of the biopsy needle in the axial plane, which is done to confirm needle position, prior to biopsy

**Fig. 13:**
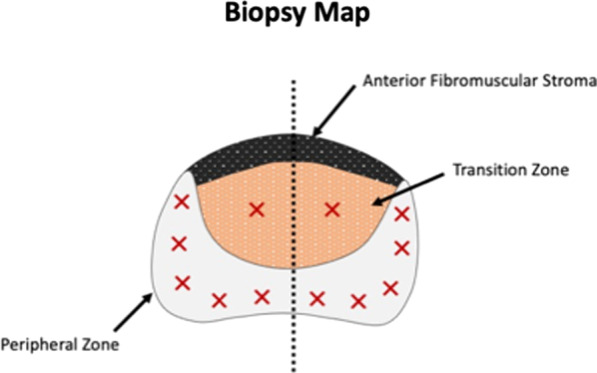
Standardised sampling biopsy map

In athletic and younger patients, the urogenital diaphragm consisting of the transverse perineal muscle, vessels and fascia may be prominent (Fig. 9) and this finding should be evaluated for on the initial ultrasound when deciding needle route to the prostate. Passing the local anaesthesia needle and introducer needle through this region, often seen approximately 1–2 cm below the levator muscle on the longitudinal view, may be painful and require additional local anaesthesia. If a prominent urogenital diaphragm is seen (Fig. 10), staying more posteriorly (Fig. 10A—red arrow) in relation to the rectal orifice will help one to avoid the urogenital diaphragm, as opposed to a more anterior location in relation to the rectum where the urogenital diaphragm is found (Fig. 10B—blue arrow pointing to the urogenital diaphragm on Fig. 10). Once the patient is adequately anaesthetised, an 11 cm 17 G introducer needle is inserted under ultrasound guidance so that its tip lies at the apex of the prostate. The longitudinal ultrasound probe transducer demonstrates the prostate in the longitudinal view. The relative mobility of the perineal skin and ischiorectal fat allows for manipulation of the co-axial needle to access all areas of the prostate. The introducer needle is passed through the levator muscle in a trajectory towards the mid-prostate gland in the anteroposterior (AP) plane (Figs. 11A, 12B) to allow one to sample around the periphery of the prostate, anteriorly and posteriorly. The introducer needle is also placed laterally on the transverse plane to ensure sampling of the peripheral gland—Figs. 11B, 12B show the biopsy needle (red arrow) located within the peripheral gland on the example. The introducer needle can then be angled to take representative samples around the periphery of the gland and transitional gland (Fig. 13).

The angle of the introducer needle may need to be repositioned during the procedure. This can happen when the gland is very big or if a lesion is located very anteriorly, posteriorly or medially. For example, if one needs to sample an anterior lesion, such as one in the anterior fibromuscular stroma, the introducer needle may need to be withdrawn from the levator muscle and passed more anteriorly through the levator muscle to sample this area (Fig. 12—blue arrow). Similarly, if the lesion is very posterior in the gland, one may need to re-position the introducer needle more posteriorly through the levator muscle to target the posterior gland specifically (Fig. 12—yellow arrow). If the introducer needle and biopsy needle are pointed posteriorly, care must be taken so that the biopsy needle does not pass outside of the prostate gland and into the rectum as this may introduce contamination into the procedure. This can be avoided by using longitudinal and axial ultrasound images while advancing the biopsy needle and locating the needle tip prior to taking the sample.

**Fig. 14:**
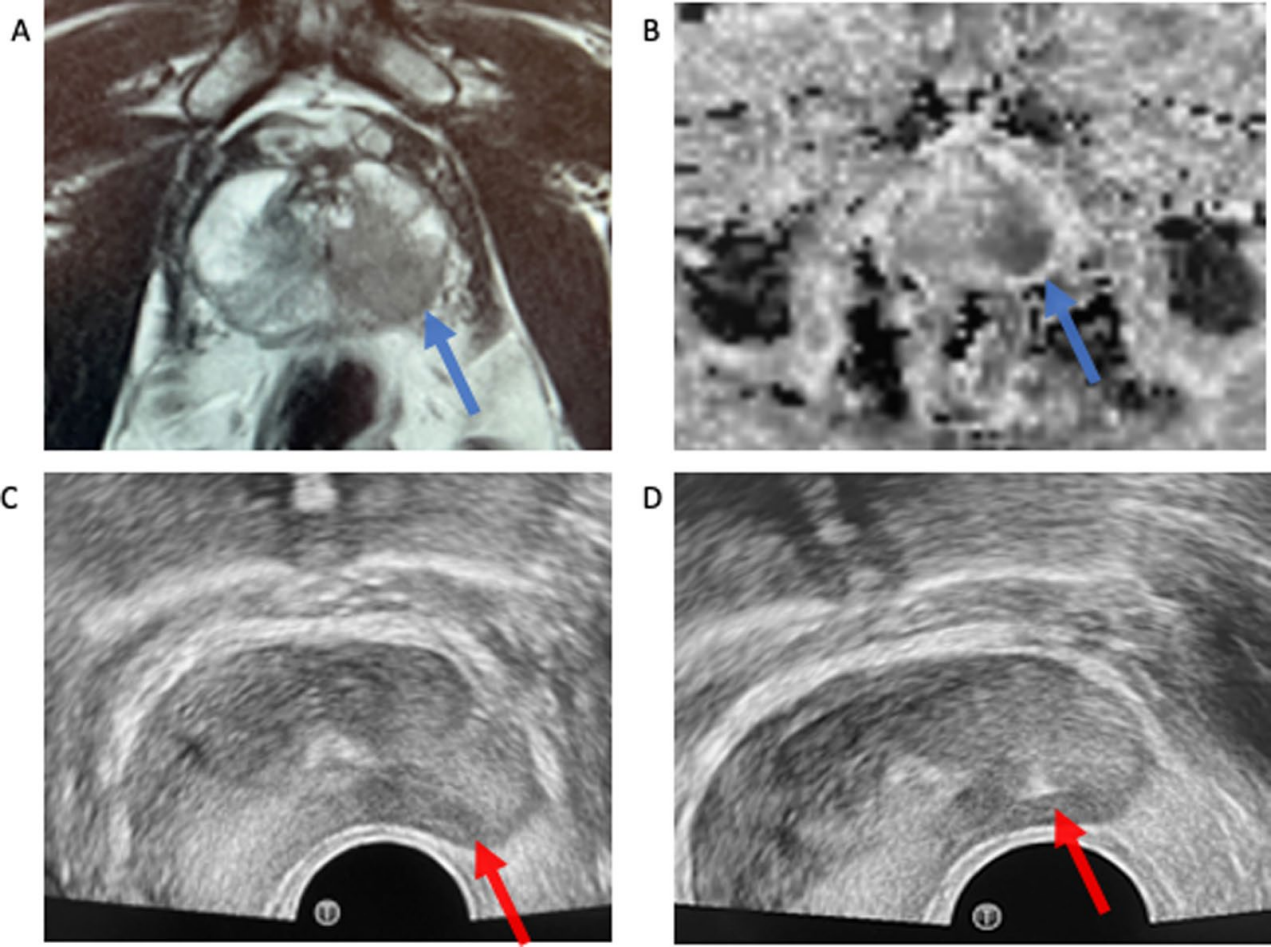
**A** and **B** show a PIRADS 5 lesion within the left mid peripheral zone on T2-weighted imaging and the associated ADC map. **C** shows the correlate identified at the time of ultrasound with **D** confirming needle position within the targeted lesion

All biopsies are then taken through the 17 G Introducer co-axial needle. Once the co-axial needle is in position, a 16-cm 18 G biopsy needle with a 2 cm tray is inserted (Fig. 11). Biopsies are acquired in a craniocaudal direction from apex to base in contrast to conventional TRUS biopsy where samples are obtained in a posterior-to-anterior direction.

**Fig. 15:**
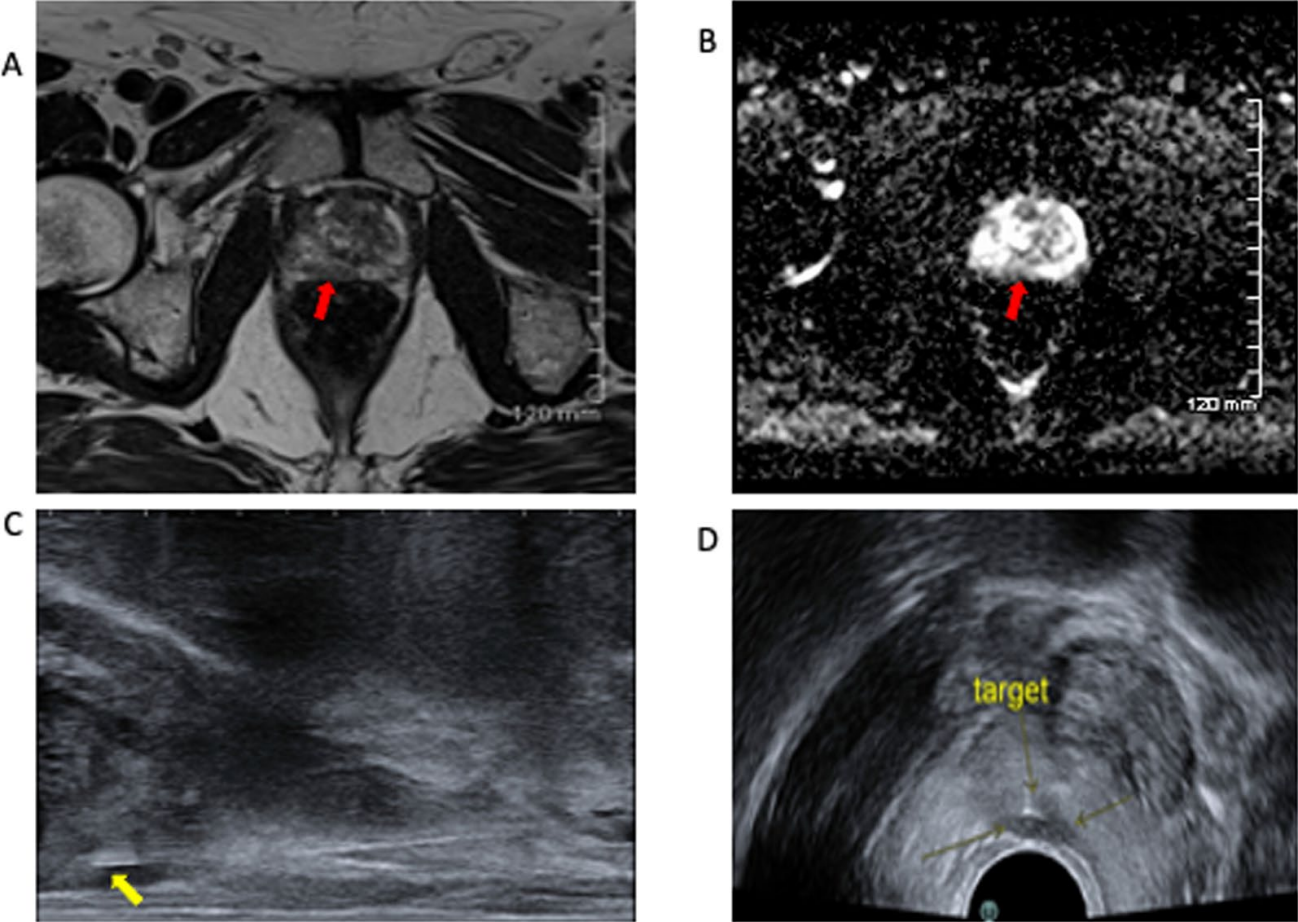
Another example of a PIRADS 5 lesion on T2-weighted imaging (**A**) and the ADC map (**B**). The needle position is confirmed to be within the lesion at time of biopsy on both sagittal images (**C**) and axial (**D**)

In our practice, transverse views are primarily used to localise the biopsy device within the desired location within the prostate gland but intermittent switching to the longitudinal transducer is frequently employed to maximise accuracy. Images of the biopsy needle can be taken for each biopsy, providing a record of the regions of the gland sampled. This is particularly useful for demonstrating accurate position of the biopsy needle in any target lesion (Figs. 14, 15). Care is taken to include samples from the apex as well as the base. This can be confirmed by switching to sagittal plane, or on axial imaging, the biopsy tray can be extended, then advanced or withdrawn to ensure the either the base of apex is included in the sample. After sampling is complete within the right gland, the left gland is then accessed via the same co-axial technique.

Targeted and non-targeted samples may be taken. Our institution has a standard systematic sampling protocol of five samples spread evenly through the left and right peripheral zones, with a further one sample of both left and right transitional zones for a total of 12 biopsy samples. Targeted biopsies are performed for PIRADS 4 and 5 lesions (Figs. 14, 15) and can be considered for PIRADS 3 lesions as per NCCP guidelines (National Cancer Control Programme). Targeted samples are labelled separately as such, including side. Of note, as biopsies are performed in a craniocaudal direction, localisation to the base, mid-gland or apex of the prostate gland is not specified and the operator must ensure that samples from various depths are obtained.

When finished, the co-axial needle and ultrasound probe are removed, and Opsite Spray bandage is used for the two puncture sites. The legs are immediately lowered. The whole procedure, including setup, takes approximately 20–30 min. The ultrasound probe is then placed in a trophon decontamination unit for disinfection. This takes 10 min and allows for preparation of the next procedure to begin.

## Procedural artefacts

**Fig. 16:**
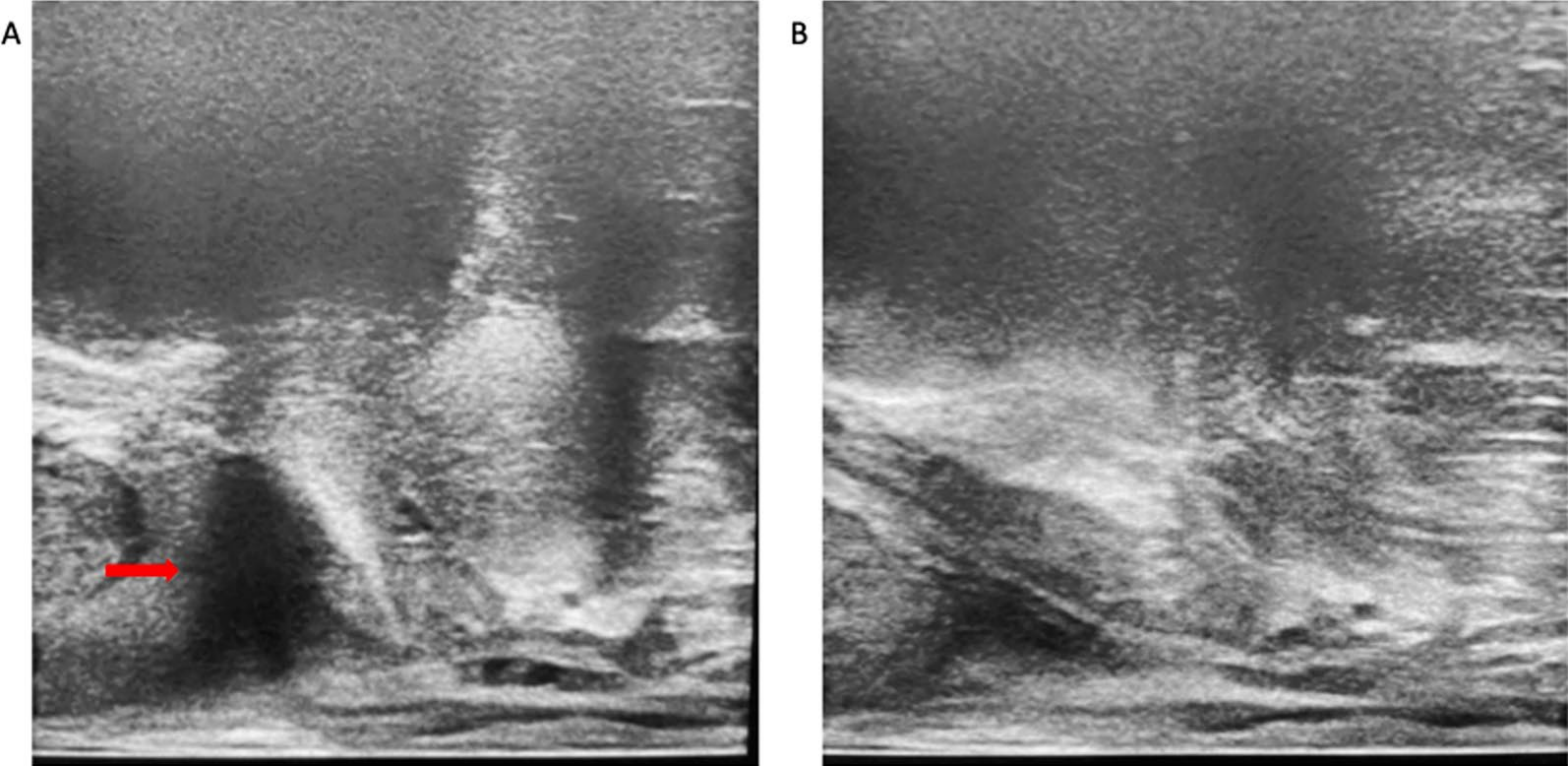
**A** shows air in the rectum (red arrow) which impedes visualisation of the prostate gland. In **B** the probe has been lifted superiorly to displace the air and improve visualisation of the gland

Air in the rectum often effects the ultrasound image which can effect procedure time and quality (Fig. 16A). The simplest way to overcome this is by displacing the air by gently moving and elevating the ultrasound probe (Fig. 16B). This brings the probe in contract with the anterior wall of the rectum, displacing the air inferiorly.

**Fig. 17:**
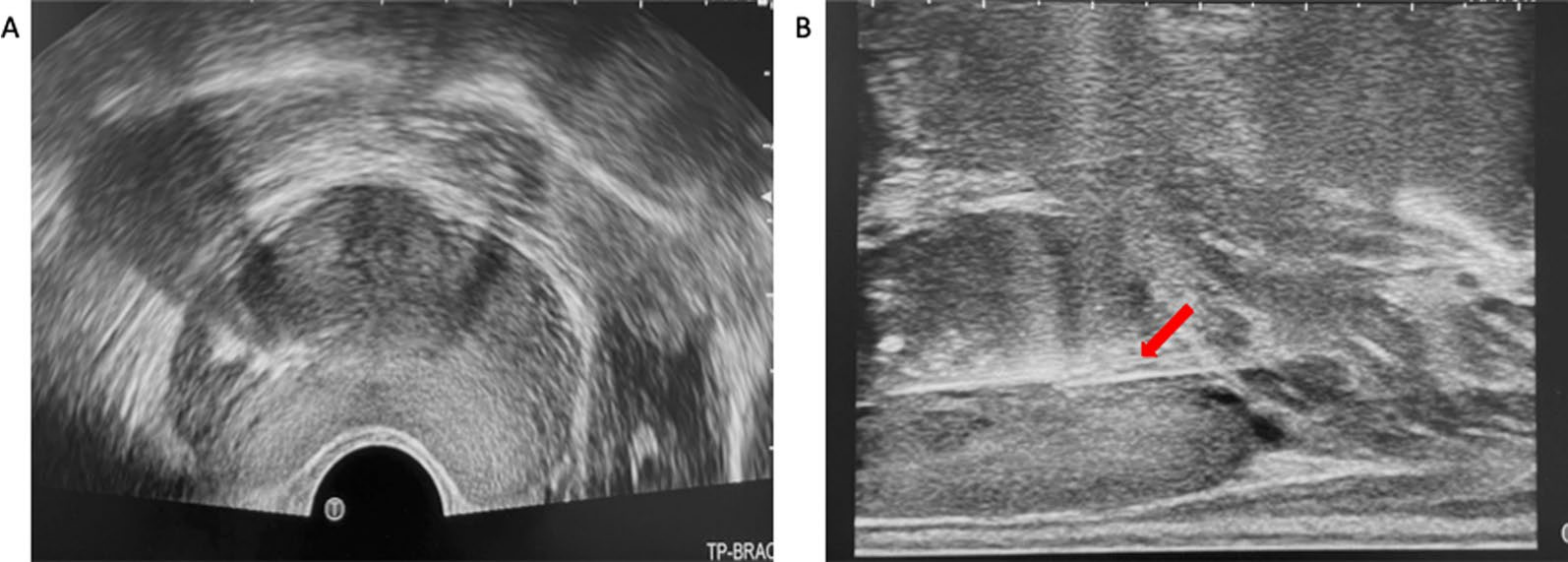
**A** shows how hyperechoic foci, likely representing a combination of air and haemorrhage, can accumulate during biopsy. By changing to a sagittal view (**B**), the needle can be seen more readily, to ensure needle visualisation prior to biopsy

Development of multiple small hyperechoic foci throughout the prostate gland is typically seen to varying degrees as the procedure progresses (Fig. 17A). These hyperechoic foci likely represent a combination of punctate intraprostatic haemorrhage and tiny locules of air introduced during needle exchange. Differentiation between these foci and the location of the biopsy needle can be challenging, particularly in transverse imaging. This can be overcome by toggling between the transverse and longitudinal planes to help locate the needle which can be better visualised on the longitudinal view in this circumstance which then allows the tip of the biopsy needle to be safe seen and accurate biopsy to be performed (Fig. 17B). For this reason, it may be helpful to biopsy targets before moving onto background non-targeted sampling biopsies.

## Post-procedural care

After the procedure, patients are taken to the radiology day ward for recovery. They are given a snack and encouraged to drink plenty of fluids. Nursing staff ensures the patient has successfully urinated prior to discharge. No further antibiotic is required. Patients are advised that they may feel perineal pain when the anaesthetic wears off and are advised to take paracetamol as required. Common post-procedural outcomes that were highlighted in the consent process are again discussed with the patient and any questions they may have are answered. They are given a letter detailing the procedure and contact details for the specialist prostate cancer nursing staff. The nurses follow up each patient with a phone call the following day to ensure they are progressing with their recovery.

## Conclusion

Transperineal Prostate Biopsy is a safer procedure than transrectal biopsy, which has been shown with multiple studies. Until recently, replacing TRUS biopsy with TP biopsy was challenging due to the need of general anaesthetic and associated operating theatre time. TP biopsy under local anaesthetic now allows for this transition to a safer and quicker procedure. Although this is a technically more challenging procedure, the procedure is well tolerated. With recent guidance from the European Association of Urology favouring the TP route [15], the time for transition is now. This paper may be used as a guide to highlight techniques which will aid in this transition.

## Abbreviations

TP biopsy: Transperineal ultrasound-guided prostate biopsy
TRUS biopsy: Transrectal ultrasound-guided prostate biopsy
